# Pathological complete response to long-course neoadjuvant alectinib in lung adenocarcinoma with EML4-ALK rearrangement: report of two cases and systematic review of case reports

**DOI:** 10.3389/fonc.2023.1120511

**Published:** 2023-06-20

**Authors:** Liang Shi, Shuhong Gao, Li Tong, Qiyi Meng, Shijie Zhou, Daping Yu, Yujie Dong, Zhe Liu

**Affiliations:** ^1^ Department of Medical Oncology, Beijing Tuberculosis and Thoracic Tumor Research Institute, Beijing Chest Hospital, Capital Medical University, Beijing, China; ^2^ Department of Thoracic Surgery, Beijing Tuberculosis and Thoracic Tumor Research Institute, Beijing Chest Hospital, Capital Medical University, Beijing, China; ^3^ Department of Pathology, Beijing Chest Hospital, Beijing Tuberculosis and Thoracic Tumor Research Institute, Capital Medical University, Beijing, China

**Keywords:** lung adenocarcinoma, ALK rearrangement, neoadjuvant therapy, alectinib, pathological complete response

## Abstract

**Objective:**

Despite the promising efficacy and tolerability of alectinib in treating advanced anaplastic lymphoma kinase (ALK) positive non-small cell lung cancer (NSCLC), the role of alectinib in neoadjuvant setting remains understudied in ALK-rearranged resectable lung cancer.

**Methods:**

Our report concerns two cases of early-stage NSCLC with complete pathologic responses to off-label use of long-course neoadjuvant alectinib. PubMed, Web of Science, and Cochrane Library were searched comprehensively for ALK-positive resectable cases with neoadjuvant alectinib. The papers were chosen following PRISMA recommendations. Seven cases from the literature and two present cases were evaluated.

**Results:**

Two cases with stage IIB (cT3N0M0) EML4-ALK lung adenocarcinoma received long-course (more than 30 weeks) of neoadjuvant alectinib followed by R0 lobectomy with the complete pathological response. In our systematic review, 74 studies were included in the original search. Application of the screening criteria resulted in 18 articles deemed eligible for full-text reading. Following the application of the exclusion criteria, out of six papers, seven cases were selected for inclusion in the final analysis and were included in the systematic review. None of the studies were included in the quantitative analysis.

**Conclusion:**

We report two cases of lung adenocarcinoma with resectable ALK-positive that achieved pCR with long-course neoadjuvant alectinib. Our cases and a systematic review of the literature support the feasibility of neoadjuvant alectinib treatment for NSCLC. However, large clinical trials must be conducted in the future to determine the treatment course and efficacy of the neoadjuvant alectinib modality.

**Systematic review registration:**

https://www.crd.york.ac.uk/PROSPERO, identifier CRD42022376804.

## Introduction

Echinoderm microtubule-associated protein-like 4-anaplastic lymphoma kinase (EML4-ALK) rearrangement, also known as the ALK-positive variant, is one of the significant driver mutations in non-small-cell lung cancer (NSCLC) and accounts for about 5% of NSCLC (1–3). This alteration leads to the uncontrolled growth and spread of the tumor cells, which is a crucial reason why ALK-positive NSCLC is more aggressive and resistant to traditional chemotherapy treatment than NSCLC without this alteration (4). In addition, ALK rearrangements are found more often in younger never smokers and in people with adenocarcinoma, the most common pathological type of NSCLC (4, 5).

We are fortunate that there have been advances in ALK tyrosine kinase inhibitor (ALK-TKI) targeted therapies for ALK-positive lung cancer over the last decade. Now, various drugs can be used for first-line treatment options in advanced lung cancer with ALK rearrangement, from the initial generation of crizotinib to the second generation of ceritinib, alectinib, and brigatinib, to the third generation of lorlatinib (6–10).

Previous studies suggest that in resectable non-small cell lung cancer, neoadjuvant chemotherapy, radiotherapy, immunotherapy, and combination therapy play an important role. Early meta-analysis suggests that neoadjuvant chemotherapy can lead to an absolute survival improvement of 5% at five years, from 40% to 45% in stage I-III resectable cases (11). In addition, the newly published CheckMate 816 study suggests that neoadjuvant chemotherapy in combination with nivolumab compared with chemotherapy alone can further improve the pathological complete response (pCR) and event-free survival (EFS) in the stage IB (≥4 cm) to IIIA resectable NSCLC patients, according to the seventh edition staging criteria of the American Joint Committee on Cancer(AJCC) (12). Based on the available evidence (12, 13), in the current eighth edition staging system of the AJCC, stage IIA-IIIA resectable NSCLC patients without EGFR mutations or ALK fusions are the target population for neoadjuvant chemotherapy in combination with immunotherapy.

For resectable NSCLC with a specific oncogenic driver positive, the published neoadjuvant study focused mainly on the epidermal growth factor receptor (EGFR) pathway. The early randomized phase 2 study of CTONG1103 showed that neoadjuvant erlotinib is more effective than neoadjuvant chemotherapy in EGFR-positive patients with stage IIIA-N2 NSCLC (14). According to previous studies, ALK-rearranged lung cancer patients have similar or worse survival outcomes than EGFR-mutated patients (15, 16). In advanced lung cancer, similar to EGFR tyrosine kinase inhibitor (EGFR-TKI) for EGFR-mutated patients, ALK-TKI has a higher objective response rate (ORR) and longer progression-free survival (PFS) for ALK-rearranged patients compared to traditional chemotherapy (6, 7), so neoadjuvant therapy for ALK-positive patients also worth exploring. However, previous neoadjuvant treatments for ALK-positive patients have mostly been clinical studies or case reports in small samples (17, 18). The ongoing phase II ALNEO trial assesses the activity of oral 8-week neoadjuvant alectinib in potentially resectable stage III ALK-positive NSCLC (any T stage with N2 or T4N0-1) (19). The other ongoing NAUTIKA1 study is a multiple cohorts perioperative trial in patients with resectable stage II-III NSCLC, which investigates neoadjuvant and adjuvant alectinib in the ALK-positive cohort (20).

Alectinib is a highly selective ALK inhibitor currently used as the front-line or second-line treatment for advanced ALK-positive NSCLC (6, 21). However, alectinib's activity as neoadjuvant therapy in resectable ALK-positive NSCLC has yet to be investigated despite promising efficacy and tolerability in treating advanced ALK-positive NSCLC.

Although several previous studies have reported cases receiving neoadjuvant treatment with alectinib (19, 22), given that there are no published results of strictly designed and implemented large trials of patients with neoadjuvant alectinib, the preoperative regimens and treatment timing of alectinib have not been well studied. Furthermore, no case has been reported or studied regarding long courses of neoadjuvant alectinib therapy. Herein, firstly, we report two cases of resectable ALK-positive lung adenocarcinoma receiving more than six months of alectinib as neoadjuvant therapy followed by radical surgical resection to achieve pathological complete responses. Subsequently, we systematically reviewed previously reported cases of neoadjuvant treatment with alectinib.

## Case presentation

### Case one

**Figure 1:**
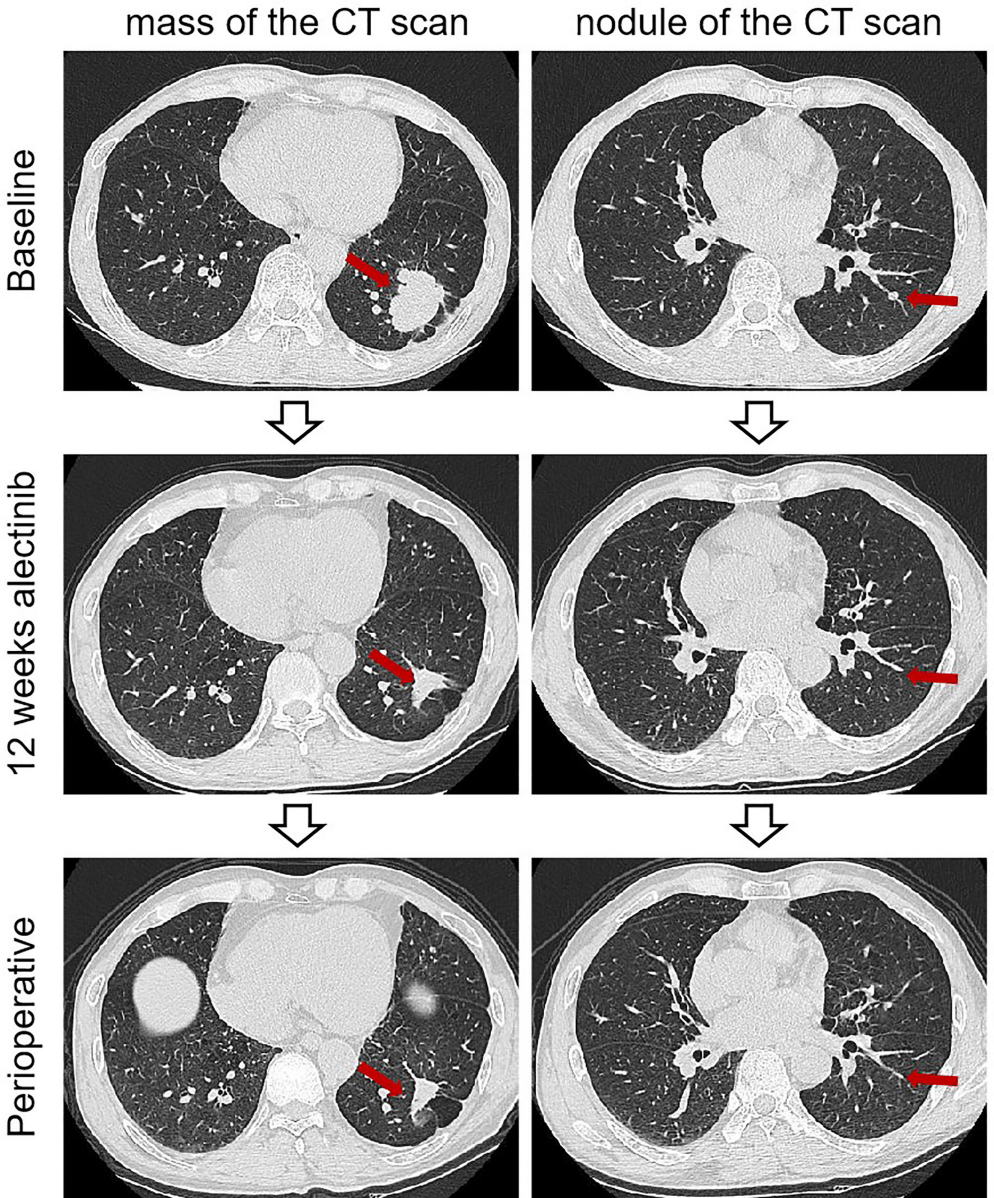
Imaging findings of case 1. The computed tomography scan of before and after neoadjuvant alectinib. CT, computed tomography.

**Figure 2:**
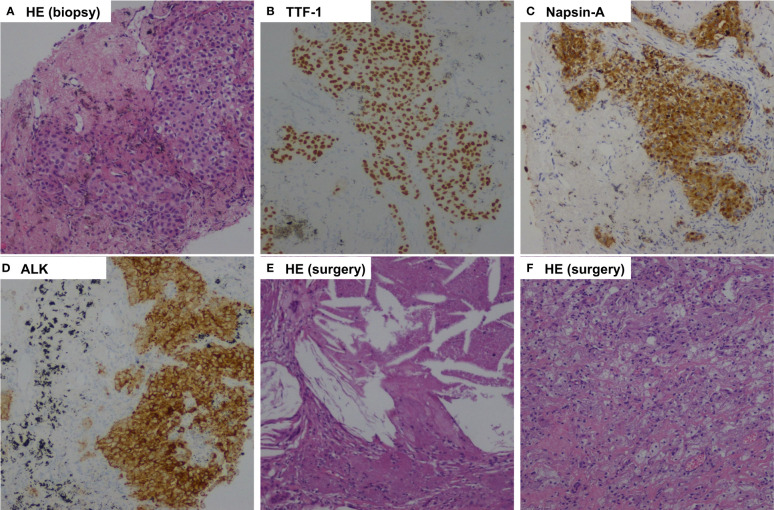
Pathologic findings of case 1. **(A)**, Percutaneous lung biopsy of the mass before treatment showed pulmonary adenocarcinoma (Hematoxylin-eosin, HE, 100x ); **(B)**, Tumor cells positive by immunohistochemistry for TTF-1 (100x); **(C)**, Tumor cells positive by immunohistochemistry for Napsin-A (100x); **(D)**, Immunohistochemical testing results (D5F3 assay) confirmed strong positivity of ALK (100x ); **(E, F)**, Postoperative pathology examination showed pathological complete response to neoadjuvant alectinib with no residual viable tumor cell. (HE, 100x ).

A 51-year-old male heavy-smoker patient presented to our hospital in July 2021 with chest pain for two months. In contrast-enhanced computerized tomography (CT) of the chest, a mass of approximately 53 mm in length was found in the left lower lobe, along with an isolated nodule of 6 mm in the same lung lobe (**Figure 1**). Serum oncological marker results showed an elevated level of carcinoembryonic antigen (CEA, 8.96 ng/ml, reference value <6 ng/ml). Fiberoptic bronchoscopy showed no significant abnormalities. The patient had a CT-guided percutaneous lung biopsy and was diagnosed with pulmonary adenocarcinoma (**Figure 2**). Immunohistochemical testing results (D5F3 assay, Ventana-Roche Diagnostics, Mannheim, Germany) confirmed anaplastic lymphoma kinase (ALK) positivity (**Figure 2**). In addition, Echinoderm microtubule-associated protein-like 4 (exon 13)-ALK (exon 20) fusion (variant 1) was detected in tumor specimen with a 60-gene panel next-generation sequencing. A whole-body bone scan, contrast-enhanced magnetic resonance imaging (MRI) scan of the head, and CT scan of the abdomen were performed to rule out distant metastases.

The patient was recommended to receive neoadjuvant alectinib therapy followed by surgical resection after a multidisciplinary discussion. Alectinib was given at 600 mg twice daily for three cycles (12 weeks) from July 15, 2021. Imaging evaluation of the efficacy showed a partial response, the patient was advised to consider receiving surgical treatment, but the patient refused surgery, continued with oral alectinib treatment, and the patient's mass continued to shrink. After 30 weeks of therapy, A CT scan was performed to assess the efficacy of neoadjuvant therapy. A partial response was achieved after neoadjuvant therapy, with 66% shrinkage of the mass (18 mm×15 mm), and the solitary nodule in the left lower lobe disappeared (**Figure 1**). Repeated serial serum CEA results showed that CEA gradually declined. Only grade 1 anemia was observed during the neoadjuvant therapy. A lobectomy of the left lower lobe and systemic lymphadectomy under a video-assisted thoracoscopic approach was successfully performed on March 1, 2022. During the surgical procedure, mild tissue adhesions were found, and no significant hilar fibrosis or significant adhesions of lymph nodes were detected. The duration of the surgery was 153 minutes, with 100 ml of intraoperative bleeding without blood transfusion. The thoracic drain was removed on postoperative day 5. The patient was discharged on postoperative day 10. Postoperative pathology shows chronic inflammation of lung tissue, more histiocytic infiltration, multinucleated giant cell reaction, and cholesterol crystals. As per IASLC multidisciplinary recommendations for pathologic assessment of lung cancer resection specimens after neoadjuvant therapy (23), the components of the primary tumor bed are 0% viable tumor, 10% necrosis, and 90% stroma, consistent with pCR (**Figure 2**). In addition, no metastatic carcinoma was seen in any lymph nodes resected in stations 4L, 5L, 7, 8, 10L, and 11L (a total of 9 lymph nodes). Postoperative pathological TNM stage down to ypT0N0M0. He continued to receive alectinib after discharge and did not report any specific discomfort at the 13-month follow-up (until April 2023). The CT scan showed the cancer had not returned at the last follow-up after the surgery. The repeated examination of the patient's CEA levels showed they were all within the normal range.

### Case two

**Figure 3:**
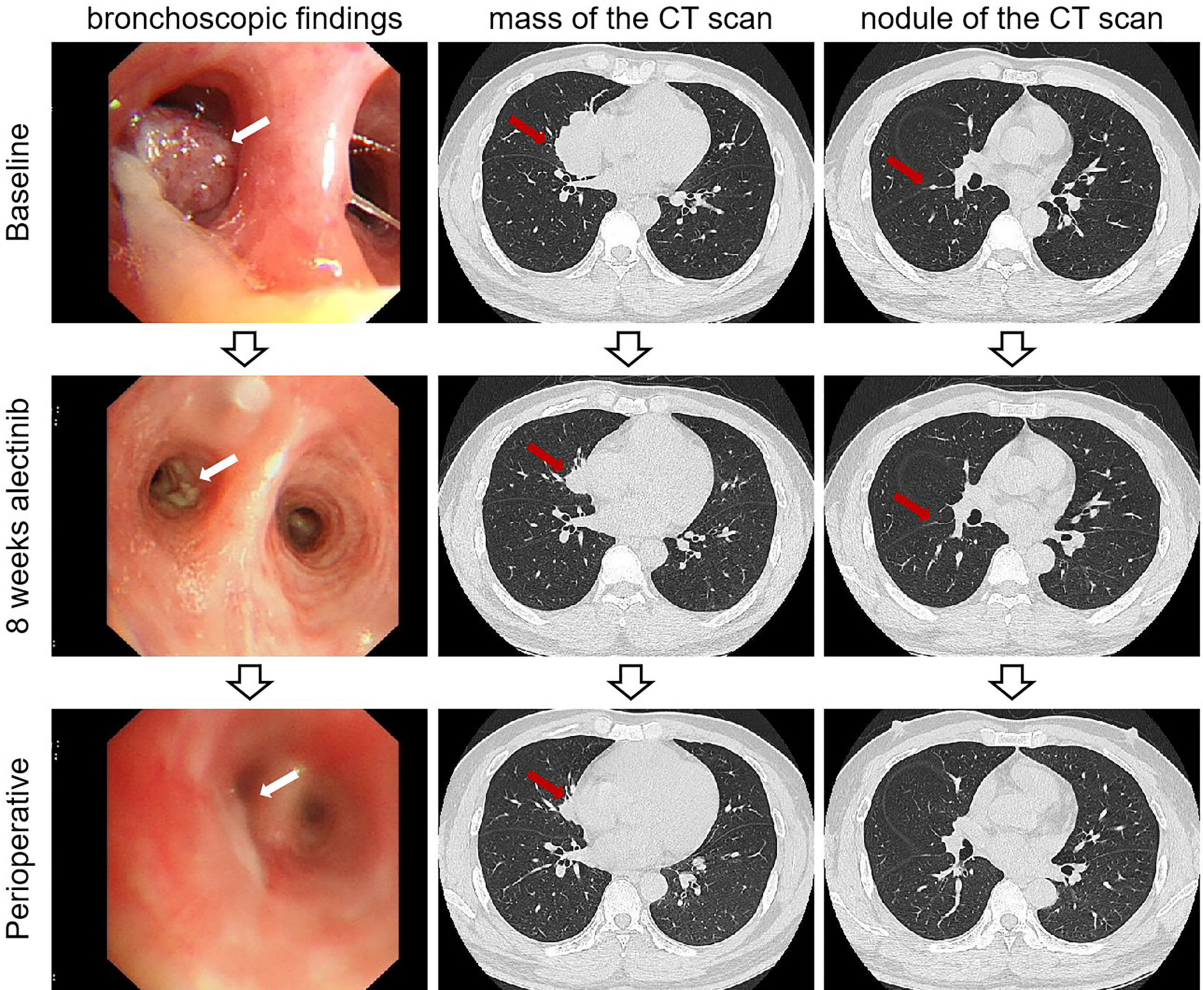
Imaging findings of case 2. The bronchoscopic findings and computed tomography scan of baseline and after neoadjuvant alectinib. CT, computed tomography.

**Figure 4:**
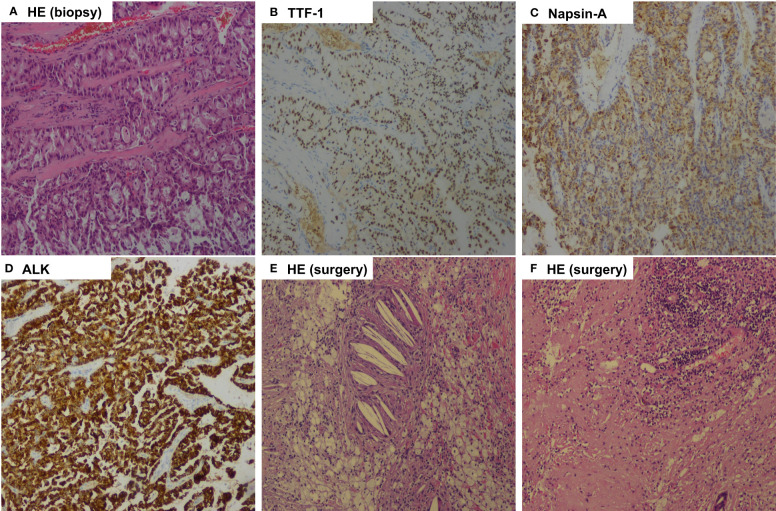
Pathologic findings of case 2. **(A)**, Bronchoscopic biopsy of the mass before treatment showed pulmonary adenocarcinoma (Hematoxylin-eosin, HE, 100x ); **(B)**, Tumor cells positive by immunohistochemistry for TTF-1 (100x); **(C)**, Tumor cells positive by immunohistochemistry for Napsin-A (100x); **(D)**, Immunohistochemical testing results (D5F3 assay) confirmed strong positivity of ALK (100x); **(E, F)**, Postoperative pathology examination showed pathological complete response to neoadjuvant alectinib with no residual viable tumor cell. (HE, 100x).

A 48-year-old man with no smoking history was referred to the hospital with an asymptomatic mass in the middle lobe of the right lung. An enhanced computed tomography scan revealed a large mass with a diameter of 58 mm and an additional nodule (5 mm) on the right interlobar pleural in the same lobe (**Figure 3**). Bronchoscopy showed a neoplasm obstructed the medial segment of the right middle lobe bronchus (**Figure 3**). Bronchoscopic biopsy pathology suggested lung adenocarcinoma, containing an acinar subtype component. Tumor cells were positive by immunohistochemistry for TTF-1 and Napsin-A (**Figure 4**). ALK fusion status was positive by immunohistochemistry with a monoclonal antibody (D5F3, Ventana-Roche Diagnostics, Mannheim, Germany) and next-generation sequencing with a 60-gene panel (Novogene Bioinformatics Technology, Beijing, China). NGS disclosed an ALK rearrangement with EML4-ALK fusion (variant 3). After a detailed staging examination, which included brain contrast-enhanced MRI, whole-body bone imaging, and contrast-enhanced CT of the chest and abdomen, no distant metastases were found.

After multi-disciplinary team consultation and acquiring informed consent from the patient, the patient began to receive treatment with neoadjuvant alectinib at 600 mg twice daily. After eight weeks of treatment with alectinib, this patient was evaluated as having a partial response (PR) by Response Evaluation Criteria in Solid Tumors (RECIST) version 1.1.

After 32 weeks of alectinib treatment, a re-evaluation showed a 70% reduction of the tumor in the right middle lobe, with no pleural nodules detected (**Figure 3**). Moreover, the patient tolerated well and only experienced grade 1 elevated aminotransferase. An R0 right middle lobectomy and systemic lymphadenectomy under a video-assisted thoracoscopic approach were performed one week after the last dose of alectinib. During surgery, moderate tissue adhesions were found during the separation of vessels and bronchi. Postoperative pathology shows chronic inflammation of resected lung tissue, fibrous tissue hyperplasia, infiltration of multinucleated giant cells, lymphocytes, and more foam cells, necrosis, and cholesterol crystals. Per IASLC 2020 methodology (23), the components of the primary tumor bed are 0% viable tumor, 25% necrosis, and 75% stroma. No metastatic carcinoma was found in the lymph nodes resected in stations 2R, 4R, 7R, 8R, 10R, 11R, 12R, and 13R (a total of 18 lymph nodes). In addition, the pathology of the resected nodule on the right interlobar pleural in the right middle lobe suggests chronic inflammation of fibrous connective tissue. Postoperative histologic examination demonstrated a complete pathological response (**Figure 4**). The patient was recommended to take alectinib for two more years after surgery and did not experience a recurrence during the twelve months of follow-up (until April 2023).

## Materials and methods

### Protocol and registration

This systematic review was reported following the Preferred Reporting Items for Systematic Reviews and Meta-Analyses (PRISMA) standard (**Table S1** in **Supplementary Materials**) (24). In addition, this systematic review protocol was registered with the international prospective register of systematic reviews (PROSPERO) online database (PROSPERO Identifier: CRD42022376804).

### Search strategy

We established our search strategy in PubMed by leveraging Medical Subject Headings (MeSH) terms for case reports and case series study types involving neoadjuvant alectinib in lung adenocarcinoma with EML4-ALK rearrangement. The generated search strategy was then transferred to the Web of Science and Cochrane Library using a Polyglot translator (25). The last search ran on 18th October 2022. The **Supplementary Materials** provide a detailed description of each database's search methodology.

### Eligibility criteria

We included case reports, case series, or letters that met all of the following criteria (1): cases with NSCLC, (2) with confirmed presence of EML4-ALK rearrangement, (3) with neoadjuvant alectinib treatment, (4) with pathological response outcome. There were no patient demographics or language restrictions, and no publication date restrictions were placed on the included studies.

### Study screening and selection

Titles and abstracts were screened for the records obtained during the literature search. Two reviewers (LS and SH-G) separately screened titles and abstracts, and consensus settled discrepancies. After that, the full texts of eligible papers were retrieved and independently double-screened, with any discrepancies being forwarded to a third reviewer (ZL).

### Data extraction

The following information was extracted from the included articles: age of the reported case, gender, smoking status, symptoms, baseline cTNM stage, EML4-ALK variant status, neoadjuvant treatment course, radiologic response, pathologic response, and adverse effects. All of the identified studies were listed by the authors, as well as the year of publication. Data extracted from each study was conducted by two reviewers independently (LS and SH-G), with any inconsistencies referred to a third reviewer (ZL). The data was summarized and compiled into an online Excel spreadsheet for the authors to access.

### Quality of studies

Case reports are biased by their nature. However, standardized techniques have been developed to evaluate the methodological quality of case reports. We utilized the Newcastle-Ottawa Scale (NOS), as modified by Murad et al., to rate the quality of the case series and reports included in the study (26). This tool evaluates the four domains of selection, ascertainment, causality, and reporting using a set of 8 questions. The tool's questions 4, 5, and 6 were omitted since they primarily apply to the kind of adverse medication events listed therein and have no bearing on our subject (26). The remaining five questions' total scores were used to categorize the remaining articles' bias risks as "high risk," "medium risk," or "low risk." We deemed case reports or case series to have a low risk of bias if they received 4 or 5 points on the quality evaluation questions. A score of 3 indicated a medium risk of bias in an article, whereas a score of less than 3 indicated a high risk of bias.

### Data analysis

Each article's data included in our systematic review was retrieved, compiled, and shown in a table. The cases were then described narratively in the text to combine and highlight the similarities and differences between them and to draw conclusions finally. We used descriptive statistics to summarize demographics and clinical characteristics due to the descriptive nature of this systematic review and the small number of cases. Continuous variables were reported using means, while dichotomous variables were reported using frequencies and percentages.

We used descriptive statistics to summarize demographics and clinical features due to the descriptive nature of this systematic review and the small number of cases. Continuous variables were reported using means or median, while dichotomous variables were reported using frequencies and percentages.

## Results

### Study selection

**Figure 5:**
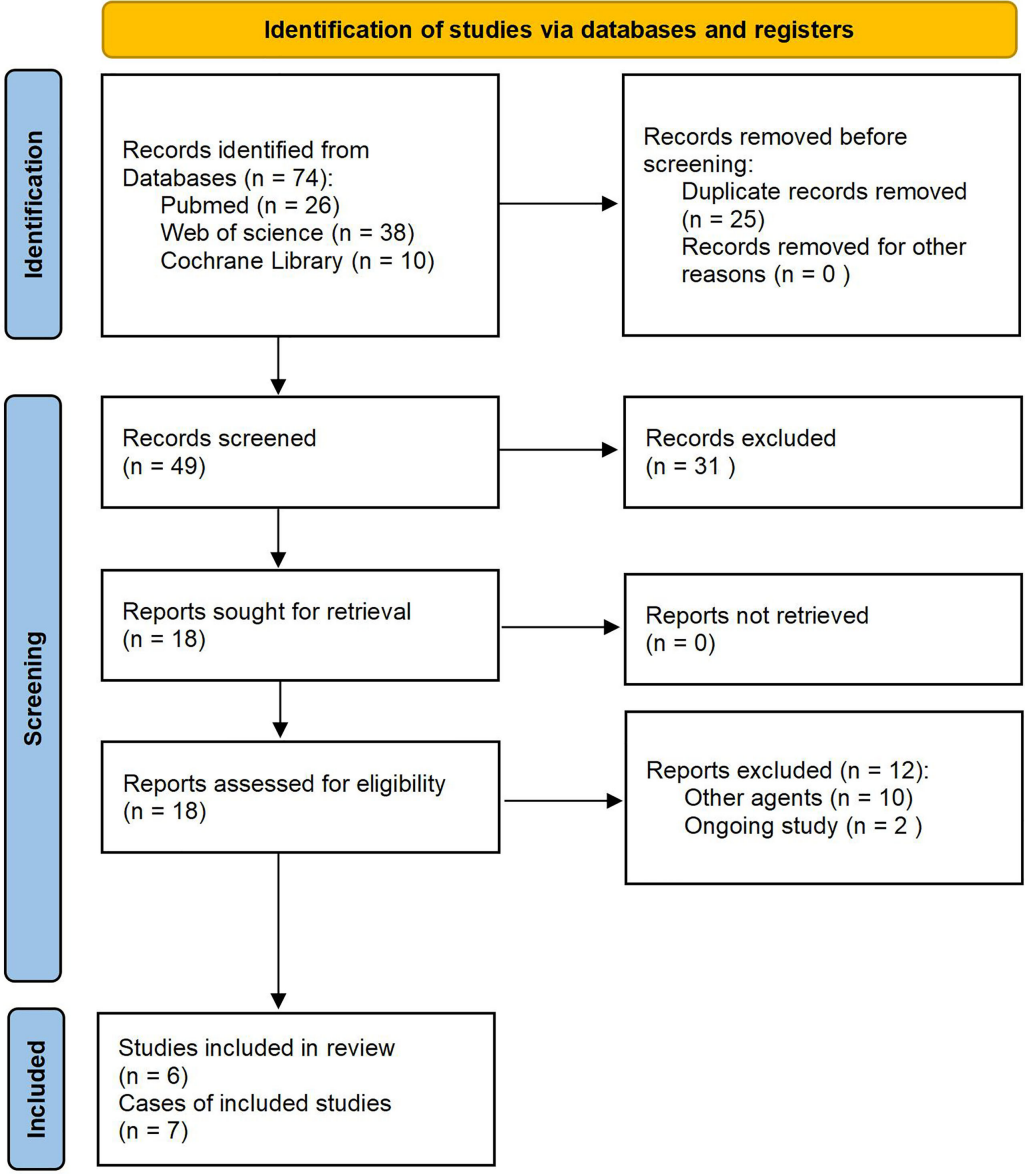
PRISMA flow diagram of the study selection process. PRISMA, Preferred Reporting Items for Systematic Reviews and Meta-Analyses.

A PRISMA flow diagram that depicts the study selection procedure is shown in **Figure 5**. First, 74 manuscripts were identified: 26 from PubMed, 38 from the Web of Science, and ten from Cochrane Library. After removing any papers that were duplicates, 49 papers remained. Of those, 31 were excluded after the title and abstract screening, leaving 18 studies for full-text screening. Next, we obtained the full text for all 18 records and screened them for eligibility. After excluding twelve articles for the reasons shown in **Figure 5**, we were left with six manuscripts, including seven cases, which we analyzed (19, 22, 27–30).

### Study and clinical characteristics of patients with neoadjuvant alectinib treatment

A summary of the characteristics is presented in **Table 1**. Of the seven previously reported cases treated with neoadjuvant alectinib, four cases were non-smokers, and three cases were former smokers. In addition, five patients were clinically staged as stage cIIIA, and two were clinically staged as stage cIIIB. Regarding the length of neoadjuvant alectinib treatment, two patients were treated for six weeks, three for eight weeks, and two for 12 weeks, with a mean of 8.6 weeks. After neoadjuvant targeted therapy, the best imaging outcome was PR in six cases, and CR was achieved in one case.

**Table 1:** Summary of reported cases receiving neoadjuvant alectinib therapy in ALK-positive lung adenocarcinoma.

Case	Author	Age(year)/Gender	Smoking status	Symptoms	Location	Baseline cTNM	EML4-ALK variant	Alectinib dosage	Treatment course	Radiologic response	Extent of resection	Pathologic response	Adverse effects	Adjuvant treatment	Follow-up
1	Zhang et al.	46/Male	None	Cough and hemoptysis	Left lower lobe	cIIIb(cT3N2M0)	variant 3	600 mg BID	8 weeks	PR	Lobectomy	non-MPR	Grade 1 constipation	NA	NA
2	Yue et al.	51/Male	Former	Asymptomatic	Right upper lobe	cIIIa(cT2N2M0)	NA	600 mg BID	6 weeks	PR	Lobectomy	non-MPR	None	Alectinib, PORT	6 months without Recurrence
3	Leonetti et al.	62/Male	None	NA	Left upper lobe	cIIIa(cT2aN2M0)	NA	600 mg BID	8 weeks	PR	Lobectomy	MPR	None	Alectinib	NA
4	Gu et al.	67/Male	None	Hoarseness	Left upper lobe	cIIIb(cT4N2M0)	NA	150 mg BID	12 weeks	CR	Lobectomy	MPR	None	NA	NA
5	Hu et al.	58/Female	None	Hemoptysis	Right lower lobe	cIIIa(cT2bN2M0)	NA	600 mg BID	8 weeks	PR	Bilobectomy	pCR	Grade 1 constipation Grade 1 erythema	Alectinib	8 months without Recurrence
6	Sentana-Lledo et al.	61/NA	Former	Asymptomatic	Left lingular lobe	cIIIa(cT1bN2M0)	variant 3	600 mg BID	6 weeks	PR	Lobectomy	pCR	NA	Alectinib	3 months without Recurrence
7	Sentana-Lledo et al.	65/NA	Former	Asymptomatic	right middle lobe	cIIIa(cT3N2M0)	variant 2	600 mg→450mg BID	12 weeks	PR	Lobectomy	MPR	Transaminases increased	Without	recurrence at 12 months mark
8	Present case 1	51/Male	Former	Chest pain	Left lower lobe	cIIb (cT3N0M0)	variant 1	600 mg BID	30 weeks	PR	Lobectomy	pCR	Grade 1 anemia	Alectinib	13 months without Recurrence
9	Present case 2	48/Male	Former	Asymptomatic	right middle lobe	cIIb (cT3N0M0)	variant 3	600 mg BID	32 weeks	PR	Lobectomy	pCR	Grade 1 transaminases increased	Alectinib	12 months without Recurrence

EML4-ALK, echinoderm microtubule-associated protein-like 4 anaplastic lymphoma kinase; PR, partial response; CR, complete remission; MPR, major pathological response; pCR, pathological complete response; NA, not available; PORT, postoperative radiotherapy.

All patients underwent lobectomy combined with mediastinal lymph node dissection and had R0 resection. Postoperative pathological results suggested pCR in 2 cases (28.6%), major pathological response (MPR) in 3 cases (42.9%), and non-MPR in 2 cases (28.6%).

We could not do a quantitative meta-analysis due to the case report or case series nature of the studies on this subject.

### Quality assessment

The quality of case reports and case series were assessed using Murad et al.’s standardized tool. According to the risk of bias score and classification rules mentioned above, five cases were scored 5, two cases were scored 4, and all cases were evaluated as low-risk of bias. A detailed quality assessment for each case is available in **Table S2** of the **Supplementary Materials**.

## Discussion

### Main findings

Our case report is the first to document a long-course of neoadjuvant alectinib treatment. Two patients with ALK-positive stage IIB NSCLC received more than six months of induction alectinib treatment and then had surgery for R0 resection after imaging evaluation showed the best shrinkage. Pathological evaluation after surgery showed that the treatment achieved pCR. In addition, there were no severe treatment-related adverse reactions (TRAEs) or severe perioperative adverse events. The patient had oral alectinib as postoperative adjuvant therapy, and follow-up showed no recurrence or metastasis.

Based on the case reports we have presented, we have conducted a systematic review of seven cases from six studies, including cases with neoadjuvant alectinib. In our systematic review, all patients with stage IIIA-IIIB NSCLC treated with a short-course of preoperative alectinib (6-12 weeks) had a postoperative pathological evaluation. Two patients had pCR, three had MPR, and two had non-MPR. There were no reported severe TRAEs. Compared with previous cases, our reported cases of long-course neoadjuvant alectinib had similar therapeutic safety and surgical feasibility. In addition, the pathological response evaluation of our reported cases reached pCR after surgery, which may bring long-term survival benefits to patients.

### Current topics in practice

One of the most critical questions is what kind of patient should receive neoadjuvant targeted therapy for ALK-positive NSCLC. In addition, the choice of therapeutic drugs and treatment cycles is another critical question.

The first neoadjuvant treatment exploration of ALK-TKI was a small sample of retrospective studies of crizotinib. In the study, all 11 patients were cases with N2 (stage IIIA or IIIB) who received crizotinib for 28-120 days before surgery. The study found that 10 out of 11 patients (91.0%) had a partial response, while one had a stable disease. Additionally, two patients (18.2%) achieved pCR (17).

With the iterations of ALK-TKI, from first-generation to third-generation drugs, we also have more available TKIs for neoadjuvant strategy against the ALK pathway. More stringent requirements are necessary for choosing drugs with higher objective response rates (ORR), remission depth, and better safety for neoadjuvant therapy, with the purpose of the neoadjuvant therapy per se. The SAKULA study showed the clinical benefit of ceritinib for neoadjuvant therapy. The study included seven patients with ALK-positive stage II-III NSCLC who received two cycles (28 days per cycle) of ceritinib induction therapy followed by surgery, with a 57% MPR and 2 cases achieving pCR (29%). However, it was worrying that all seven patients had treatment interruptions due to adverse events, and five cases experienced dose downregulation (31).

A series of studies on the front-line alectinib in advanced NSCLC showed that alectinib has a higher ORR and a greater tumor remission depth than crizotinib and may be more valuable for neoadjuvant therapy (9, 32, 33). Therefore, the neoadjuvant exploration of alectinib is also reasonably expected. The earliest case report suggests that alectinib for neoadjuvant therapy resulted in tumor shrinkage. A 46-year-old male with clinical stage IIIB (cT3N2M0) lung adenocarcinoma received neoadjuvant alectinib at 600 mg twice daily for two cycles (56 days), and imaging results after two cycles of treatment evaluated PR, with tumor shrinkage of 47%. After induction of alectinib, the TNM stage was downstaged as IB (ypT1aN0M0) (22). The cases of other previous small samples also suggested the value of alectinib in neoadjuvant therapy. However, neoadjuvant targeted therapy data is relatively scarce, and clinicians primarily rely on their experience. There is a need for more research to improve current data.

Most reported cases in our review were locally advanced patients with initial clinical stage III. However, the two cases we reported were stage IIB patients with suspicious intrapulmonary metastatic nodules (T3) at initial evaluation and recommended to receive induction therapy after multidisciplinary discussion. Notably, our patients had a smaller tumor load and earlier clinical stage than previously reported cases, which may have contributed to the eventual postoperative pathological evaluation to achieve pCR.

Additionally, studies included in our review had administered targeted therapy for 2-3 months, while few cases had received long cycles of alectinib preoperatively. Our reported cases, however, received more than six months of alectinib each, and both patients underwent surgery after achieving the best objective response when the tumor reached maximum remission. The earlier initial clinical stage is another critical factor contributing to our cases' postoperative pCR. However, this hypothesis requires further confirmation through strictly designed clinical trials.

The current NAUTIKA1 trial involving the neoadjuvant treatment of alectinib is enrolling patients with resectable stage II, IIIA, or selective IIIB (T3N2 only) ALK-positive NSCLC. These patients will receive alectinib induction therapy for eight weeks, followed by surgery (20). The other ALNEO study, which includes cases of potentially resectable stage III (any T with N2, T4N0-1) NSCLC, will also receive alectinib induction therapy for eight weeks (19, 34). These prospective clinical trials deserve our expectations.

### Strength and weaknesses

Our study is the first to focus on long-course neoadjuvant alectinib in lung adenocarcinoma with the EML4-ALK variant. It aims to integrate all available cases from the literature in a systematic review with a standardized quality appraisal. While our review is a crucial starting point for understanding the preoperative ALK-TKI therapy strategy, its findings need to be expanded upon by more rigorous studies in prospective clinical trials. However, our narrative synthesis of case reports has several weaknesses, including subjectivity and a lack of detailed patient information and long-term follow-up data to judge overall survival accurately. Additionally, the small sample size of seven cases prevented a thorough quantitative synthesis, and generalizability is limited due to missing cases and publication bias. Furthermore, our analysis of neoadjuvant therapy findings was restricted by the lack of control over reported pathological response results.

## Conclusion

We present two cases of resectable ALK-positive lung adenocarcinoma that had a pCR following long-course neoadjuvant alectinib treatment. Our cases, along with a systematic review, show that neoadjuvant alectinib treatment is a feasible option for NSCLC. However, large clinical trials are necessary in order to determine the treatment course and efficacy of neoadjuvant alectinib.

## Data availability statement

The raw data supporting the conclusions of this article will be made available by the authors, without undue reservation.

## Ethics statement

This study was approved by the institutional review board (IRB)/ethics committee of Beijing Chest Hospital, Capital Medical University.

## Author contributions

ZL, LS, and SG contributed to the study's conception, data interpretation, and manuscript writing. LS, SG, LT, QM, SZ, DY, YD, and ZL analyzed the patient data. All authors contributed to the article and approved the submitted version.
