# Supplementary material for: Pathological complete response to long-course neoadjuvant alectinib in lung adenocarcinoma with EML4-ALK rearrangement: report of two cases and systematic review of case reports
Source: Front Oncol. 2023 Jun 20;13:1120511. doi: 10.3389/fonc.2023.1120511 (PMC10318538; doi:10.3389/fonc.2023.1120511)
Supplement: Supplementary file 1 [file Table_1.docx]

## Supplementary material

Pathological Complete Response to Long-Course Neoadjuvant Alectinib in Lung Adenocarcinoma with EML4-ALK Rearrangement : Report of Two Cases and Systematic Review of Case Reports

Liang Shi, Shuhong Gao, Li Tong, Qiyi Meng, Shijie Zhou, Daping Yu, Yujie Dong, and Zhe Liu

#### Table of Contents

Table S1: Prisma 2020 checklist

Search strategy

Table S2: Quality assessment

#### Table S1: Prisma 2020 checklist

| **Section and Topic** | **Item #** | **Checklist item** | **Location where item is reported** |
| --- | --- | --- | --- |
| **TITLE** | | |  |
| Title | 1 | Identify the report as a systematic review. | Page 1 |
| **ABSTRACT** | | |  |
| Abstract | 2 | See the PRISMA 2020 for Abstracts checklist. | Page 2 |
| **INTRODUCTION** | | |  |
| Rationale | 3 | Describe the rationale for the review in the context of existing knowledge. | Page 3-4 |
| Objectives | 4 | Provide an explicit statement of the objective(s) or question(s) the review addresses. | Page 4 |
| **METHODS** | | |  |
| Eligibility criteria | 5 | Specify the inclusion and exclusion criteria for the review and how studies were grouped for the syntheses. | Page 7 |
| Information sources | 6 | Specify all databases, registers, websites, organisations, reference lists and other sources searched or consulted to identify studies. Specify the date when each source was last searched or consulted. | Page 6-7 Supplementary  material |
| Search strategy | 7 | Present the full search strategies for all databases, registers and websites, including any filters and limits used. | Supplementary  material |
| Selection process | 8 | Specify the methods used to decide whether a study met the inclusion criteria of the review, including how many reviewers screened each record and each report retrieved, whether they worked independently, and if applicable, details of automation tools used in the process. | Page 7  Figure 5 |
| Data collection process | 9 | Specify the methods used to collect data from reports, including how many reviewers collected data from each report, whether they worked independently, any processes for obtaining or confirming data from study investigators, and if applicable, details of automation tools used in the process. | Page 7-8 |
| Data items | 10a | List and define all outcomes for which data were sought. Specify whether all results that were compatible with each outcome domain in each study were sought (e.g. for all measures, time points, analyses), and if not, the methods used to decide which results to collect. | Page 7-8 |
|  | 10b | List and define all other variables for which data were sought (e.g. participant and intervention characteristics, funding sources). Describe any assumptions made about any missing or unclear information. | Page 7-8 |
| Study risk of bias assessment | 11 | Specify the methods used to assess risk of bias in the included studies, including details of the tool(s) used, how many reviewers assessed each study and whether they worked independently, and if applicable, details of automation tools used in the process. | Page 8 |
| Effect measures | 12 | Specify for each outcome the effect measure(s) (e.g. risk ratio, mean difference) used in the synthesis or presentation of results. | Page 8 |
| Synthesis methods | 13a | Describe the processes used to decide which studies were eligible for each synthesis (e.g. tabulating the study intervention characteristics and comparing against the planned groups for each synthesis (item #5)). | Not applicable |
|  | 13b | Describe any methods required to prepare the data for presentation or synthesis, such as handling of missing summary statistics, or data conversions. | Not applicable |
|  | 13c | Describe any methods used to tabulate or visually display results of individual studies and syntheses. | Page 8 |
|  | 13d | Describe any methods used to synthesize results and provide a rationale for the choice(s). If meta-analysis was performed, describe the model(s), method(s) to identify the presence and extent of statistical heterogeneity, and software package(s) used. | Page 8 |
|  | 13e | Describe any methods used to explore possible causes of heterogeneity among study results (e.g. subgroup analysis, meta-regression). | Not applicable |
|  | 13f | Describe any sensitivity analyses conducted to assess robustness of the synthesized results. | Not applicable |
| Reporting bias assessment | 14 | Describe any methods used to assess risk of bias due to missing results in a synthesis (arising from reporting biases). | Not applicable |
| Certainty assessment | 15 | Describe any methods used to assess certainty (or confidence) in the body of evidence for an outcome. | Not applicable |
| **RESULTS** | | |  |
| Study selection | 16a | Describe the results of the search and selection process, from the number of records identified in the search to the number of studies included in the review, ideally using a flow diagram. | Page 9  Figure 5 |
|  | 16b | Cite studies that might appear to meet the inclusion criteria, but which were excluded, and explain why they were excluded. | Page 9  Figure 5 |
| Study characteristics | 17 | Cite each included study and present its characteristics. | Page 9  Table 1 |
| Risk of bias in studies | 18 | Present assessments of risk of bias for each included study. | Page 9-10 Supplementary  material |
| Results of individual studies | 19 | For all outcomes, present, for each study: (a) summary statistics for each group (where appropriate) and (b) an effect estimate and its precision (e.g. confidence/credible interval), ideally using structured tables or plots. | Not applicable |
| Results of syntheses | 20a | For each synthesis, briefly summarise the characteristics and risk of bias among contributing studies. | Page 9  Table 1 |
|  | 20b | Present results of all statistical syntheses conducted. If meta-analysis was done, present for each the summary estimate and its precision (e.g. confidence/credible interval) and measures of statistical heterogeneity. If comparing groups, describe the direction of the effect. | Not applicable |
|  | 20c | Present results of all investigations of possible causes of heterogeneity among study results. | Not applicable |
|  | 20d | Present results of all sensitivity analyses conducted to assess the robustness of the synthesized results. | Not applicable |
| Reporting biases | 21 | Present assessments of risk of bias due to missing results (arising from reporting biases) for each synthesis assessed. | Not applicable |
| Certainty of evidence | 22 | Present assessments of certainty (or confidence) in the body of evidence for each outcome assessed. | Not applicable |
| **DISCUSSION** | | |  |
| Discussion | 23a | Provide a general interpretation of the results in the context of other evidence. | Page 10-12 |
|  | 23b | Discuss any limitations of the evidence included in the review. | Page 12-13 |
|  | 23c | Discuss any limitations of the review processes used. | Page 12-13 |
|  | 23d | Discuss implications of the results for practice, policy, and future research. | Page 13 |
| **OTHER INFORMATION** | | |  |
| Registration and protocol | 24a | Provide registration information for the review, including register name and registration number, or state that the review was not registered. | Page 6 |
|  | 24b | Indicate where the review protocol can be accessed, or state that a protocol was not prepared. | Page 6 |
|  | 24c | Describe and explain any amendments to information provided at registration or in the protocol. | Page 6 |
| Support | 25 | Describe sources of financial or non-financial support for the review, and the role of the funders or sponsors in the review. | Page 14 |
| Competing interests | 26 | Declare any competing interests of review authors. | Page 14 |
| Availability of data, code and other materials | 27 | Report which of the following are publicly available and where they can be found: template data collection forms; data extracted from included studies; data used for all analyses; analytic code; any other materials used in the review. | Page 13 |

#### Search strategy

**Pubmed: Final search run on 18/11/2022**

"carcinoma, non small cell lung"[Mesh] OR "adenocarcinoma of lung"[Mesh] OR NSCLC[tiab] OR "lung adenocarcinoma"[tiab]

AND

"anaplastic lymphoma kinase"[Mesh] OR "anaplastic lymphoma kinase"[tiab] OR ALK[tiab]

AND

alectinib[nm] OR alectinib[tiab] OR Crizotinib[Mesh] OR Crizotinib[tiab] OR ceritinib[nm] OR ceritinib[tiab] OR brigatinib[nm] OR brigatinib[tiab] OR ensartinib[nm] OR ensartinib[tiab] OR lorlatinib[nm] OR lorlatinib[tiab]

AND

"neoadjuvant therapy"[Mesh] OR neoadjuvant[tiab] OR preoperative[tiab] OR resectable[tiab] OR operable[tiab]

**Web of science: Final search run on 18/11/2022**

"carcinoma, non small cell lung" OR "adenocarcinoma of lung" OR NSCLC OR "lung adenocarcinoma"

AND

"anaplastic lymphoma kinase" OR "anaplastic lymphoma kinase" OR ALK

AND

alectinib OR crizotinib OR ceritinib OR brigatinib OR ensartinib OR lorlatinib

AND

"neoadjuvant therapy" OR neoadjuvant OR preoperative OR resectable OR operable

**Cochrane Library: Final search run on 18/11/2022**

[mh "carcinoma, non small cell lung"] OR [mh "adenocarcinoma of lung"] OR NSCLC:ti,ab OR "lung adenocarcinoma":ti,ab

AND

[mh "anaplastic lymphoma kinase"] OR "anaplastic lymphoma kinase":ti,ab OR ALK:ti,ab

AND

alectinib:kw OR alectinib:ti,ab OR [mh Crizotinib] OR Crizotinib:ti,ab OR ceritinib:kw OR ceritinib:ti,ab OR brigatinib:kw OR brigatinib:ti,ab OR ensartinib:kw OR ensartinib:ti,ab OR lorlatinib:kw OR lorlatinib:ti,ab

AND

[mh "neoadjuvant therapy"] OR neoadjuvant:ti,ab OR preoperative:ti,ab OR resectable:ti,ab OR operable:ti,ab

#### Table S2: Quality assessment following Murad et al.’s modified Newcastle–Ottawa Scale (NOS)

| Domains | Leading explanatory questions | Included cases | | | | | | |
| --- | --- | --- | --- | --- | --- | --- | --- | --- |
|  |  | Case 1 | Case 2 | Case 3 | Case 4 | Case 5 | Case 6 | Case 7 |
| Selection | 1. Does the patient(s) represent(s) the whole experience of the investigator (centre) or is the selection method unclear to the extent that other patients with similar presentation may not have been reported? | Yes | Yes | Yes | Yes | Yes | NO | NO |
| Ascertainment | 2. Was the exposure adequately ascertained? | Yes | Yes | Yes | Yes | Yes | Yes | Yes |
|  | 3. Was the outcome adequately ascertained? | Yes | Yes | Yes | Yes | Yes | Yes | Yes |
| Causality* | 4. Were other alternative causes that may explain the observation ruled out? | NA | NA | NA | NA | NA | NA | NA |
|  | 5. Was there a challenge/rechallenge phenomenon? | NA | NA | NA | NA | NA | NA | NA |
|  | 6. Was there a dose–response effect? | NA | NA | NA | NA | NA | NA | NA |
|  | 7. Was follow-up long enough for outcomes to occur? | Yes | Yes | Yes | Yes | Yes | Yes | Yes |
| Reporting | 8. Is the case(s) described with sufficient details to allow other investigators to replicate the research or to allow practitioners make inferences related to their own practice? | Yes | Yes | Yes | Yes | Yes | Yes | Yes |
| Risk of bais | | Low risk | Low risk | Low risk | Low risk | Low risk | Low risk | Low risk |

NA, not appliable.

*Questions 4,5 and 6 in the tool were left out because they are mostly relevant to cases of adverse drug events as described by the tool and do not relate to our topic.
